# Detecting causal relationship of non-floodplain wetland hydrologic connectivity using convergent cross mapping

**DOI:** 10.1038/s41598-023-44071-0

**Published:** 2023-10-11

**Authors:** Sangchul Lee, Byeongwon Lee, Junga Lee, Jihoon Song, Gregory W. McCarty

**Affiliations:** 1 Department of Environmental Engineering, University of Seoul, Dongdaemun-gu, Seoul, 02504 Republic of Korea; 2 Division of Environmental Science & Ecological Engineering, College of Life Sciences & Biotechnology, Korea University, Seoul, 02841 Republic of Korea; 3 Ojeong Resilience Institute, Korea University, Seoul, 02841 Republic of Korea; 4 USDA-ARS, Hydrology and Remote Sensing Laboratory, Beltsville, MD 20705 USA

**Keywords:** Environmental sciences, Hydrology

## Abstract

The hydrologic connectivity of non-floodplain wetlands (*NFWs*) with downstream water (*DW*) has gained increased importance, but connectivity via groundwater (*GW*) is largely unknown owing to the high complexity of hydrological processes and climatic seasonality. In this study, a causal inference method, convergent cross mapping (CCM), was applied to detect the hydrologic causality between upland *NFW* and *DW* through *GW*. CCM is a nonlinear inference method for detecting causal relationships among environmental variables with weak or moderate coupling in nonlinear dynamical systems. We assumed that causation would exist when the following conditions were observed: (1) the presence of two direct causal (*NFW* → *GW* and *GW* → *DW*) and one indirect causal (*NFW* → *DW*) relationship; (2) a nonexistent opposite causal relationship (*DW* → *NFW*); (3) the two direct causations with shorter lag times relative to indirect causation; and (4) similar patterns not observed with pseudo *DW.* The water levels monitored by a well and piezometer represented *NFW* and *GW* measurements, respectively, and the *DW* was indicated by the baseflow at the outlet of the drainage area, including *NFW*. To elucidate causality, the *DW* taken at the adjacent drainage area with similar climatic seasonality was also tested as pseudo *DW*. The CCM results showed that the water flow from *NFW* to *GW* and then *DW* was only present, and any opposite flows did not exist. In addition, direct causations had shorter lag time than indirect causation, and 3-day lag time was shown between *NFW* and *DW*. Interestingly, the results with pseudo *DW* did not show any lagged interactions, indicating non-causation. These results provide the signals for the hydrologic connectivity of *NFW* and *DW* with *GW.* Therefore, this study would support the importance of *NFW* protection and management.

## Introduction

Non-floodplain wetlands (*NFWs*) enclosed by uplands without surface runoff outlets^1^ provide hydrologic, biological, chemical, and ecological benefits to landscapes^2,3^. Thus, understanding *NFW* hydrologic connectivity with adjacent or distant water bodies through surface runoff or subsurface flow including groundwater (*GW*) is important for demonstrating their key roles in landscapes^4^. Water mediating the transport of matter, energy, and organisms within or between elements of the hydrologic cycle refers to hydrologic connectivity^5^. The degree, type, and frequency of *NFW* connectivity (i.e., hydrologic connectivity between *NFW* and other components) differ by geomorphic characteristics^6^. For example, surface water connectivity is prevalent in the Prairie Pothole region owing to limited underground hydraulic conductivity^6^, whereas *GW* connectivity between *NFWs* and other landscape components prevails on the Coastal Plain of the Chesapeake Bay Watershed (CBW)^7,8^.

Hydrologic connectivity between *NFW*s and *DW*s is a function of surface runoff and *GW*, and the major diver varies depending on the landscape and climatic characteristics^1,9^. Surface runoff connectivity is often measured using in-situ observations on the Coastal Plain of the CBW^10,11^. However, demonstrating *GW* connectivity using observational data is extremely challenging because of the inherent uncertainty in *GW* connectivity, which is characterized as a combination of nonlinear behaviors driven by interactions among climatic inputs, hydrogeologic characteristics, and human intervention^3,12^. Uncertainty regarding *GW* connectivity is further complicated by climatic seasonality (e.g., evapotranspiration [*ET*] and precipitation [*P*]). Climatic seasonality often drives landscape hydrology, leading to similar temporal dynamics of landscape components^7^.

On the coastal plain of the CBW, the *NFW* water budget increases with *P* and *GW* inflow but decreases with *ET* and *GW* outflow. When the water table is higher or lower than the bottom of *NFW* during the wet and dry seasons, *GW* inflow to and outflow from *NFW*s occur, respectively^13^. Surface inflow and outflow also affect *NFW* dynamics; however, the impact of surface runoff is mostly observed during heavy rainfall events^7^. Thus, *NFW* connectivity via surface water has been determined in this region after rainfall events or wet seasons^10,11^. In addition, *NFW* connectivity with *DW* via *GW* has been reported in this region, owing to subsurface permeable conditions that lead to the strong interactions between *NFW* and *GW*^14–17^. However, *GW* connectivity is speculated based on regional characteristics and the modeling approach; strong evidence in this regard is still lacking in this region.

**Figure 1:**
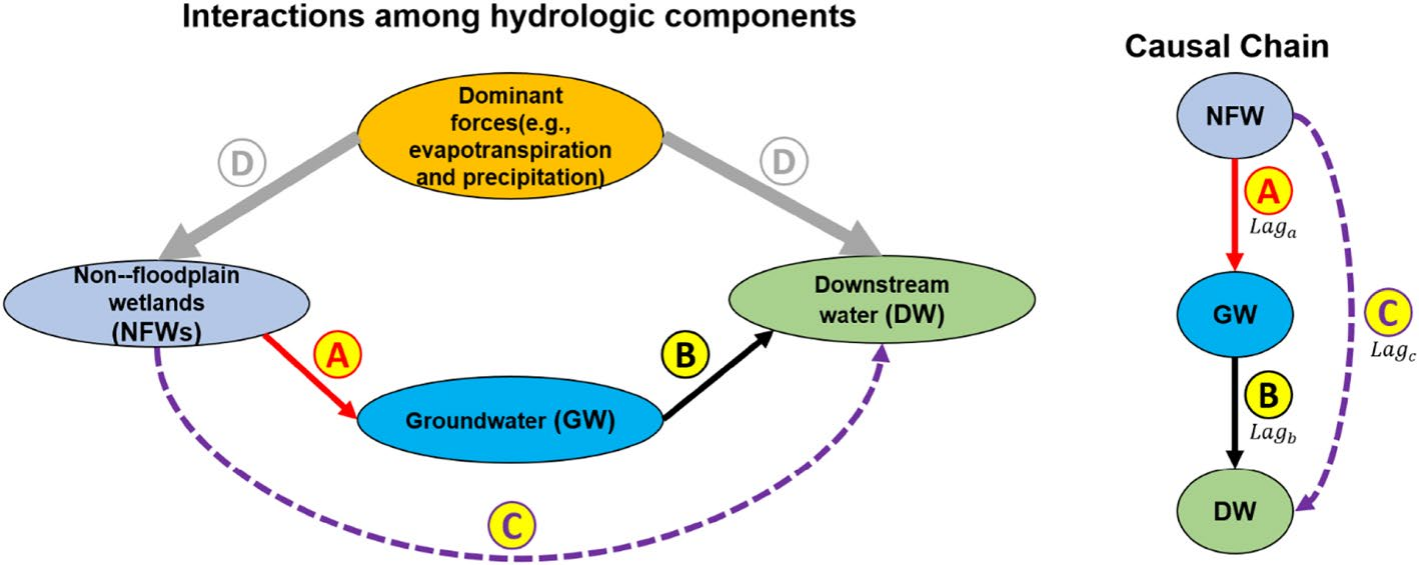
Schematic diagram showing interactions among *NFWs*, *GW*, *DW*, and climatic seasonality (left) and their causal chain (right). The figure is partially adopted from Pyzoha et al.^19^. “A”, “B”, and “C” indicate flow from *NFW* to *GW*, *GW* to *DW*, and *NFW* to *DW*, respectively. D is a causal variable shared by *NFW* and *DW*, resulting in similar patterns*.* In the causal chain, Lag_a_, Lag_b_, and Lag_c_ stand for the lag for flow from *NFW* to *GW*, from *GW* to *DW*, and from *NFW* to *DW*, respectively. The figure was generated by the MS Office PowerPoint.

The major driving forces of the regional water budget on the CBW coastal plain are *P* and *ET*. When *P* is greater or lower than ET, the water budget of the landscape components, including *NFWs* increases or decreases, respectively. Landscape water storage is strongly dependent on wetlands in this region, which are characterized by low-gradient topography and poorly drained soils^18^. These conditions collectively lead to similar dynamics of *NFW* and *DW*, but their differences are observed after rainfall events for a short period^7^. To demonstrate *GW* connectivity between *NFW* and *DW*, the three components should exhibit a chain of causal relationships (represented as “cause” → “effect”) with different lag times (Fig. 1): *NFW* → *GW* with Lag_a_, *GW* → *DW* with Lag_b_, and *NFW* → *DW* with Lag_c_. Any changes in *NFW*s will be reflected in *GW* and later in *DW*s along a hydrologic gradient due to lag times. A chain of causal relationships with *NFW* → *GW* and *GW* → *DW* have a direct causal relationship, while *NFW* indirectly affects *DW*. Thus, Lag_c_ is equal to or greater than the summation of Lag_a_ and Lag_b_ (i.e., Lag_c_ ≥ Lag_a_ + Lag_b_). In contrast, the responses of *NFW* and *DW* to climatic seasonality might be simultaneous (i.e., no lag time) when the effects in both *NFW* and *DW* are linked to a shared cause (i.e., climatic seasonality). The causal relationships between *NFW*, *GW*, and *DW* with different lag times can be indicative of *GW* connectivity. Thus, identifying *GW* connectivity requires a metric that detects the causality between two variables with time-delayed interactions within nonlinear systems.

Convergent cross mapping (CCM) is a novel approach for detecting causality in nonlinear systems^20,21^. This causal inference method is unique because it can detect causal relationships between two variables with weak or moderate coupling. CCM has been successfully tested to demonstrate causal relationships between two time-series datasets of environmental systems: temperature impacts on greenhouse gases^22^, invading species and soil nitrate^23^, between summer precipitation and aboveground biomass^23^, population dynamics of anchovies and sardines^24^, soil moisture impacts on precipitation^25^, hydrologic connectivity between two reservoirs^26^, and interactions between hydrologic and climatic variables^27^, and surface and groundwater relationship^28^. Extended CCM has also been used for distinguishing between uni- and bi-directional flow and detecting causal chains between entities with varying degrees of lagged behaviors^21^. Extended CCM can quantify whether two entities causally interact with each other (i.e., bi-directional) or whether only one entity affects the other entity (i.e., unidirectional)^21^.

In this study, we employed extended CCM to demonstrate the hydrologic connectivity of *NFW* with *DW* through *GW* and quantify the time delay in this causal relationships in the Greensboro watershed (GBW) within CBW in the USA^7^. We speculated that if *NFW* has any hydrologic connectivity with *DW* via *GW*, the following would be observed from CCM analysis: (1) two direct causality (*NFW* → *GW* and *GW* → *DW*) and one indirect causality (*NFW* → *DW*) would be present; (2) the opposite direction of causality would not appear, and (3) direct causality would have shorter lag time than indirect causality. Because of the high uncertainty in the causation between *GW* and *DW*, we considered pseudo *DW* (*DW*_adj_) adjacent to the *DW* located at the outlet of the watershed, including the *NFW* (*DW*_org_). It was also assumed that causality would be observed between *NFW* and *DW*_org_ but not between *NFW* and *DW*_adj_.

## Materials and methods

### Study area and data

**Figure 2:**
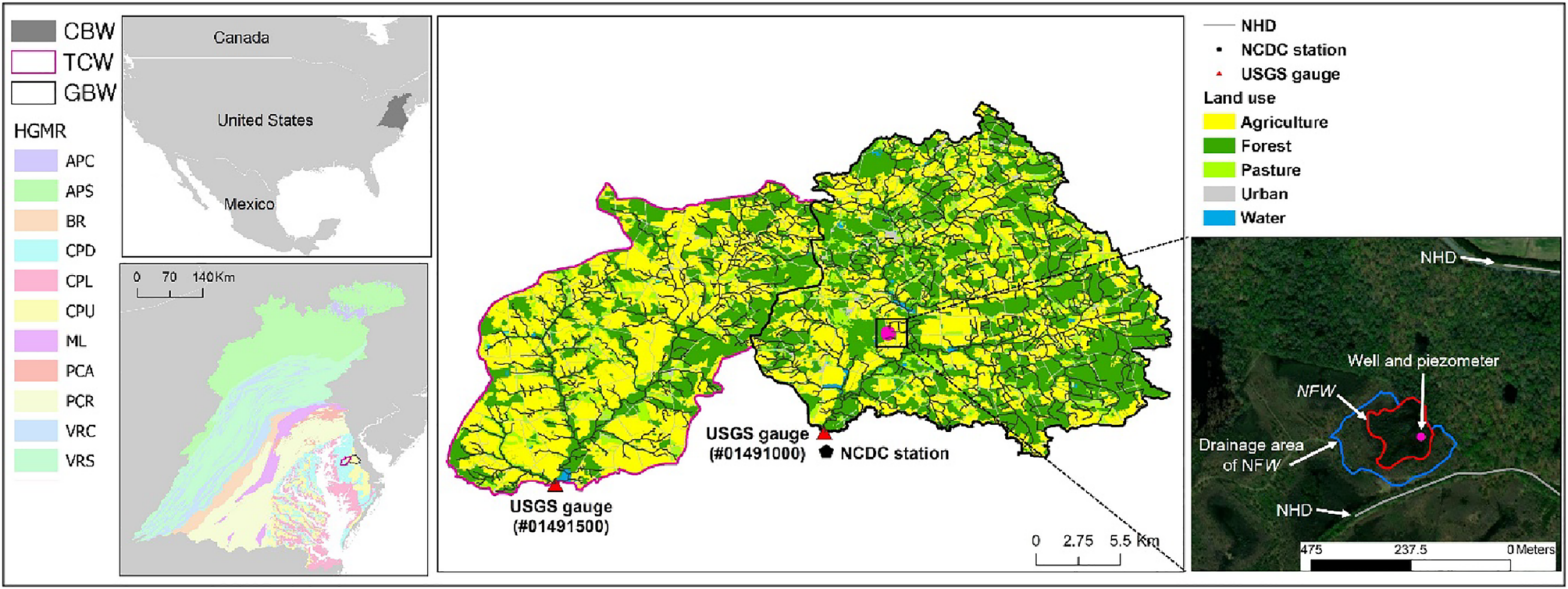
The spatial location of the studied wetland. CBW indicates the chesapeake bay watershed. GBW (Greensboro watershed) and TCW (Tuckahoe Creek watershed) are the drainage areas of the USGS gauge stations #01491000 and #01491500, respectively. NHD stands for the National Hydrography Dataset. The location of well and piezometer was further zoomed-in in Fig. S1 of the Supplementary Material and the data source used in this figure is listed in Table S1 of the Supplementary Material. The description of the hydrogeomorphic region (HGMR) is available in Table S2 of the Supplementary Material. The map was generated by ArcMap 10.7.

The *NFW* is located within the GBW, which is the drainage area of the U.S. Geological Survey (USGS) gauge station #01491000, on the coastal plain of the CBW (Fig. 2). The CBW is divided into 11 hydrogeomorphic regions (HGMRs) based on the rock type and physiographic province^17^. The study area is included in the coastal plain upland (CPU) with high precipitation infiltration into shallow aquifer due to well-drained soils and flat topography^17^. The CPU is also underlaid with unconsolidated sediments with high permeability, leading to large groundwater discharge to streams^17^.

The sizes of *NFW* and its drainage area are 1 ha and 3.1 ha, respectively; and the drainage area is dominated by forest^7^. The nearest streams indicated by the USGS high-resolution National Hydrography Dataset (NHD) is 0.1 km from *NFW* (Fig. 2). The “Wetland” component of the U.S. Department of Agriculture Conservation Effects Assessment Project (CEAP‐Wetlands) implemented a well and piezometer to explore the surface water level and groundwater level of *NFW* in this region, respectively^29^. A PVC pipe with a 2.54 cm diameter was utilized to construct a well and piezometer, with well screens being positioned over the whole length of the wells and the lower 30 cm of the piezometers. The well was utilized to directly measure the height of the water column, whereas the piezometer was employed to assess the pressure of the groundwater exerted by the water column. The well was equipped with pressure transducers (Campbell Scientific CS451, Campbell Scientific, Logan, Utah, United States), while the piezometer was physically linked to the data logger (Campbell Scientific CR1000). To see the interactions between the surface and groundwater, a well and piezometer were installed side by side (Fig. S1 of the Supplementary Material) since different soil conditions under the bottom of *NFW* might not correctly capture their interactions^30^.

The studied NFW was monitored from January 2016 to December 2019 (Table 1). The water levels were continuously collected by a well and piezometer at every 15-min, respectively (Fig. 2). The well and piezometer in the *NFW* was installed to 0.9 and 3.0 m below the wetland bottom, respectively, and they were spatially close each other^30^. Following similar previous studies^7,30^, this study assumed the water levels monitored by a well and piezometer indicated *NFW* and *GW*, respectively.

**Table 1:** Descriptions of the studied *NFW* and its monitoring.

Attributes	Descriptions			
Location	39.053 (Latitude), − 75.748 (Longitude)			
Monitoring period	January 1st, 2016 to December 31st, 2019			
Monitoring range	0.8 and 3 m below the wetland bottom (well and piezometer, respectively)			
Surface level (m)	min	max	std	Avg
	− 0.90	0.44	0.31	0.14
Groundwater level (m)	min	max	std	Avg
	− 1.38	0.32	0.37	− 0.07
Distance to *DW* (km)	7.1			
Monitoring samples (day)	*NFW*: 1259, *GW*: 1461			
Missing samples (day)	*NFW*: 177, *GW*: 0			
Area (ha)	1.0			

The location indicates the specific points of wells and piezometers installed. Non-floodplain wetland (*NFW*) and groundwater (*GW*) are defined as water levels measured by wells and piezometers, respectively, in this study. *DW* indicates downstream water.

Baseflow from streamflow collected from the USGS gauge station was used to represent *DW*_org_, and USGS gauge station #01491500 was also prepared for *DW*_adj_ (Fig. 2). Both *DW*_org_ and *DW*_adj_ were spatially adjacent, and thus they similarly responded to regional climatic seasonality. *DW*_org_ was hydrologically downstream of *NFW* based on the watershed boundary. Baseflow separation was calculated using the EcoHydRology package^31^ in the R programming environment. The digital filter separation described by Nathan and McMahon^32^ was used in this package, and the default settings were applied in our study.

15-min data were aggregated into daily values for the CCM analysis. The *NFW* measurements included 177 missing samples (January 11th–July 6th, 2017), and data incompleteness could cause errors in the CCM analysis. The missing samples were filled with simulations from the process-based model developed to predict the water level of *NFW*^33^. The process-based model was modified to simulate the hydrology of *NFW,* and the model successfully predicted the daily water level of the *NFW* studied with high accuracy with the R^2^ value of 0.84–0.88 and further detailed results are provided in Junyu et al.^33^.

### Convergent cross mapping

In this study, we introduced CCM, a method suggested by Sugihara et al.^20^ and extended by Ye et al.^21^, to detect causal relationships between variables of interest. CCM is a handy and robust numerical tool to determine causal influence when the time series of two variables are available. The theoretical basis of this approach comes from Takens’ theorem: when “x” (cause) influences “y” (effect) in dynamical systems, the value of “x” can be retrieved from the value of “y”. One critical process is to determine whether the accuracy of the reconstruction of “x” from “y” increases as the number of *L* (library vectors) of “y” increases. As the number of *L* means the number of different time points used for the reconstruction. In other words, it tests whether the inclusion of “y” values from longer periods reconstructs “x” better. The degree of how accurately “y” estimates “x”, called cross map skill (*p*), is determined based on Pearson’s correlation between observed and estimated “x”^20^. Most importantly, this test allows screening of spurious correlational relationships. If “x” and “y” have a spurious correlational relationship, an increase in *L* does not result in a more accurate reconstruction of “x”, because the correlation is only due to the short-time synchronization of the two variables. In CCM, this relationship between x and y is represented as “y xmap x” that means x is estimated from y. In this study, we stated “y (effect) xmap x (cause)” as “x → (affects) y” to better indicate the relationship between cause and effect. This study also tested the significance of CCM results (*p*-value < 0.01) to identify spurious causation using the method introduced in Bonotto et al.^28^ and Ye et al.^34^. The test included assessing whether the cross-map skill at full library size is significantly greater than the highest lagged cross-correlation and surrogate time series. The *p*-value was computed as below:

1 $$p{\text{-}}value=(n+1)/(k+1),$$

where *k* is the total number of surrogates and *n* is the number of replicates with greater cross-map skill than the actual value.

The extension and enhancement of CCM by Ye et al.^34^ solved one of the weaknesses of the original version: overwhelming influence from “x” on “y” may lead to successful CCM in both directions (meaning that “y” also affects “x”), although this is not the case. This weakness, called ‘generalized synchrony,’ occurs when “y” is almost only affected by “x”, and thus, “x” and “y” act as one system. To address this issue, Ye et al.^34^ considered different lag times. Causal relationships signify that there exists a chronological order between the two variables. Therefore, if the causal relationship between “x” and “y” is unidirectional (here only “x” affects “y”), the lag time found in CCM recreating “x” from “y” will be negative, while the time lag found in CCM recreating “y” from “x” will be positive. The optimal time lag is decided when the cross map skill is largest. The negative optimal lag time of “x (cause)” → “y (effect)” indicates the response of “y” to any changes in “x” will appear after the optimal lag time. The zero optimal lag time of “x → y” denotes “y” promptly responds to “x” without any lag time, and the positive optimal lag time of “x → y” means “x (cause)” responds to “y (effect)” with the lag time, indicating illogical causation.

We used the rEDM package developed by Ye et al.^34^ to implement CCM processes in R. The processes involved the reconstruction of individual system states using a time series to generate the joint state, for the estimation of one variable from another. Individual system states were reconstructed by the time-delay embedding approach, which represents the delay coordinates of each system state. The optimal embedding dimension (*E*) of each system state was calculated through the rEDM package in R^34^.

## Results

### Temporal dynamics of non-floodplain wetland (*NFW*), groundwater (*GW*), and downstream water (*DW*)

**Figure 3:**
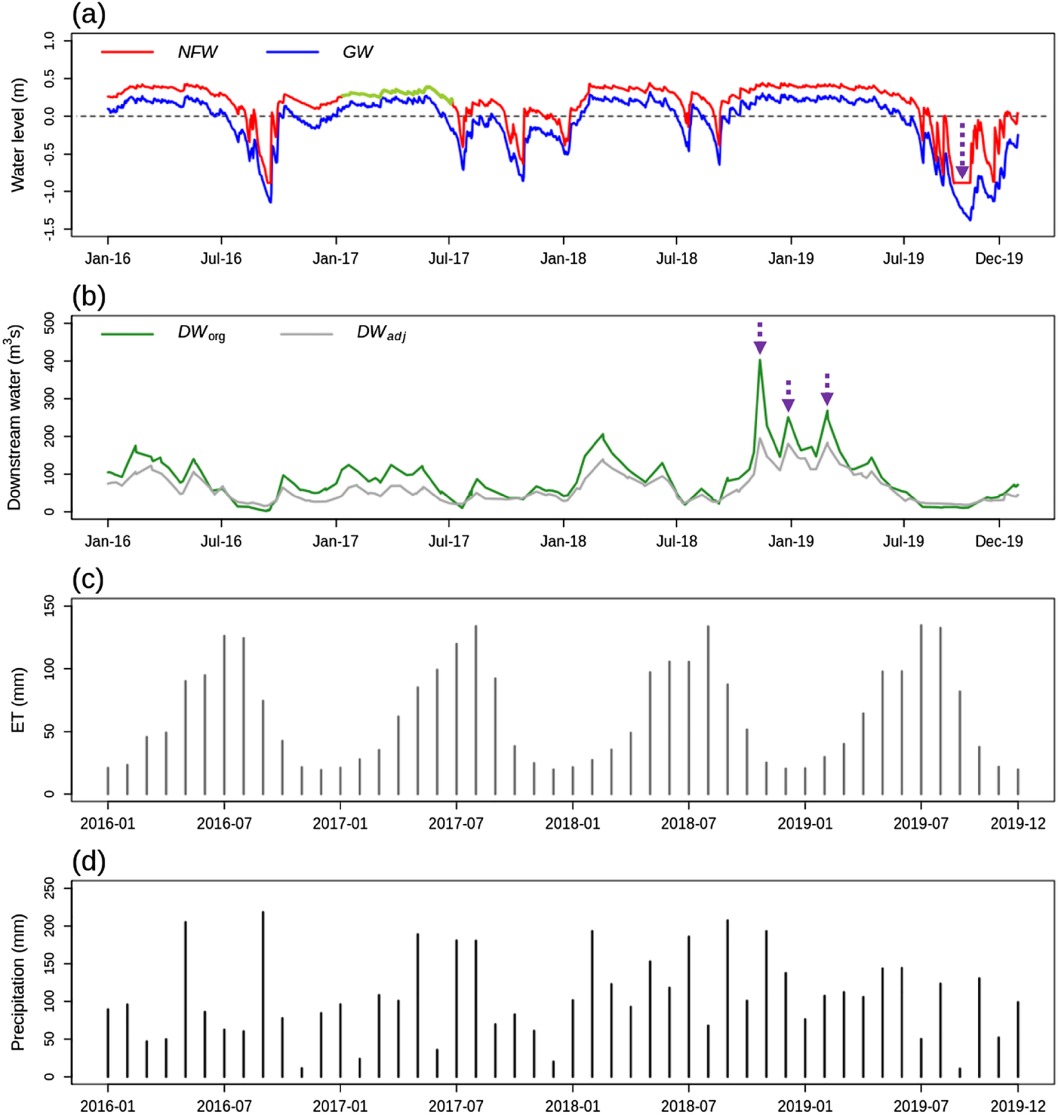
(**a**) Daily time series of non-floodplain wetlands (*NFW*) and groundwater (*GW*), (**b**) daily time series of two downstream waters (*DWs*), (**c**) monthly time series of evapotranspiration (ET), and (**d**) monthly time series of precipitation. The yellow-green line in (**a**) indicates the predicted *NFW* by the process-based model^33^. *DW*_org_ and *DW*_adj_ are baseflow derived from streamflow measured at USGS gauge stations #01491000 and #01491500, respectively. The figure was generated by the R 3.6.1 program.

The daily time series of *NFW*, *GW*, *DW*_org_, and *DW*_adj_ are shown in Fig. 3a,b. Overall, the temporal dynamics of *NFW*, *GW*, *DW*_org_, and *DW*_adj_ were highly similar in response to the seasonal trends of this area, with high and low water balance during the winter and summer seasons, respectively. *NFW* and *GW* ranged from − 0.9 to 0.4 m and from − 1.4 to 0.3 m, respectively, while the variation ranges of *DW*_org_ and *DW*_adj_ were 2–403 (m^3^/s) and 15–195 (m^3^/s), respectively. *DW*_org_ and *DW*_adj_ showed similar temporal dynamics (Fig. 3b). Monthly variations in ET were high during summer months (June, July, and August) and low during winter months (December, January, and February) and the monthly pattern of precipitation was overall uniform over the course of the year, indicating seasonality in this region (Fig. 3c,d).

*NFW* and *GW* dynamics did not comply with several peaks of *DW* owing to the vertical limits in the monitoring range of *NFW* and *GW* (dotted vertical purple arrows in Fig. 3b). When the water storage of *NFW* was filled by heavy rainfall, the fill-spill dynamics of *NFW* frequently occurred^10,11^. Regarding the configuration of a well and piezometer (Fig. S2 of the Supplementary Material) the maximum upper water level of *NFW* and *GW* is the same as the depth of the *NFW* while the monitoring range of *DW* had no limit, leading to different dynamics between *NFW/GW* and *DW*. The monitoring lower limit also caused the flat level of *NFW* during the summer of 2019 (dotted vertical purple arrow in Fig. 3a). In addition, *DW* is the summation of intra-watershed processes and thus the behavior of *DW* might be different from *NFW* and *GW* that are the tiny landscape component.

### Embedding dimension and nonlinearity

To apply the CCM method, the embedding dimension (*E*) should be determined. The embedding dimension (*E*) is the number of time steps used for the prediction. Following previous studies, the optimal *E* values for *NFW*, *GW*, and *DW* were computed using a simplex projection (Table 2)^24,25^. Nonlinearity was identified by observing the best prediction when different combinations of the degree of nonlinearity (*θ*) and embedding dimension (*E*) were tested. When the best prediction was shown with *θ* > 0, the time-series variable was nonlinear. The nonlinearity was assessed by S-map with the embedding dimension (*E,* 1, 2,…, 10) and the degree of nonlinearity (*θ,* 0, 0.25, 0.5,…, 0).

**Table 2:** The optimal embedding dimensions (*E*) and degree of nonlinearity (*θ*) of time-series observations.

Attributes	*E*	Nonlinearity
*NFW*	3	*θ* = 3.0, E = 4
*GW*	2	*θ* = 4.0, E = 3
*DW*_org_	3	*θ* = 0.75, E = 3
*DW*_adj_	2	*θ* = 8, E = 10

### Differentiation of causation from non-causal relationships

**Figure 4:**
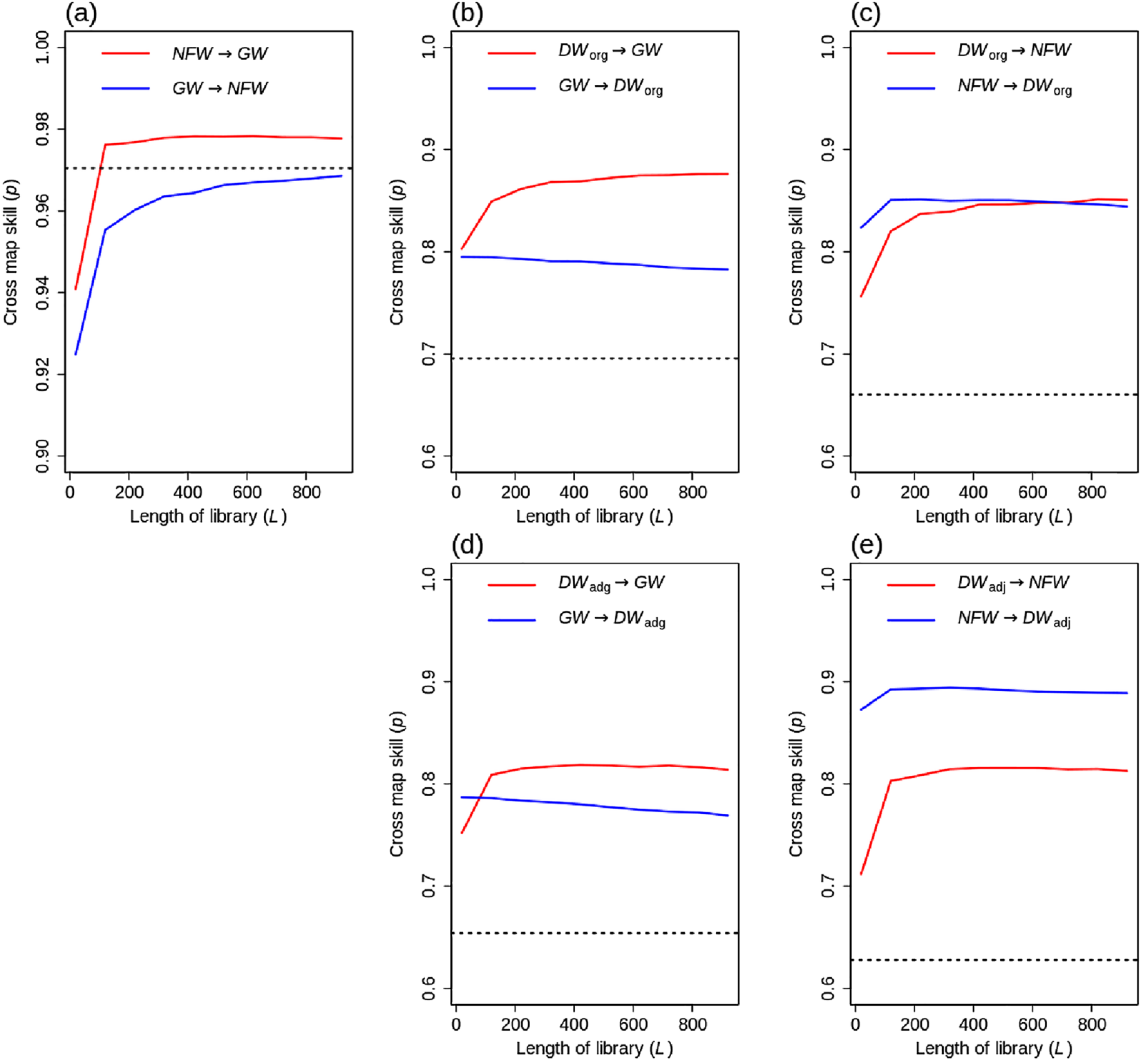
Cross map skill (*p*) of observed and estimated values as a function of the length of the library (*L*): (**a**) *NFW* and *GW*, (**b**) *GW* and *DW*_org_, (**c**) *NFW* and *DW*_org_, (**d**) *GW* and *DW*_adj_, and (**e**) *GW* and *DW*_adj_. The dotted horizontal line is the highest lagged cross-correlation. x → y indicates x affects y. *NFW* and *GW* indicate non-floodplain wetland and groundwater, respectively. *DW*_org_ and *DW*_adj_ are baseflow derived from streamflow measured at USGS gauge stations #01491000 and #01491500, respectively. The figure was generated by the R 3.6.1 program.

In the CCM method, we attempted to differentiate causation from non-causal correlations between observed and estimated variables: *NFW* → *GW*, *GW* → *DW*, and *NFW* → *DW*, using cross map skill. Figure 4 shows the cross map skill (*p*) between the observed and estimated variables with different library lengths (*L*). Interestingly, overall relationships had high cross map skill (*p*) with a longer library length (*L*) with the significant results (*p-*value < 0.01). The causality was detected at *NFW* → *GW*, which was driven by the vertical water flow from surface to groundwater. The high cross map skill (*p*) of *GW* → *NFW* was likely due to the seasonal flow from upland to *NFW* via *GW* in this region^7^. The causality results between precipitation and hydrologic variables (e.g., *NFW*, *GW*, and *DW*_*org*_) showed precipitation exhibited a causal relationship with three hydrologic variables (*p*-value < 0.01, Fig. S3 of the Supplementary Material). This finding suggested that the seasonal lateral movement of *GW* to *NFW* could be substantiated by the causative link between precipitation and these hydrologic variables.

However, the significant test showed that the cross map skill of *GW* → *NFW* at full library size was lower than the highest lagged cross-correlation, indicating non-causation (Fig. 4a). The cross map skill (*p*) of *GW* → *DW*_org_ and *DW*_adj_ was high and remained unchanged regardless of the length of the library (*L*). *DW*_org_ and *DW*_adj_ represents the combination results of hydrological landscape components. A tiny component (*GW*) could have minimal impacts on *DW*_org_ and *DW*_adj_ and thus the cross map skill (*p*) of *GW* → *DW*_org_ and *DW*_adj_ did not consistently increase with an increase in the length of the library (*L*). In contrast, *DW*_org_ and *DW*_adj_ complied with seasonal dynamics indicated by dominant driving forces (e.g., *P* and *ET*), leading to a relatively high value of the cross map skill (*p*) of *DW*_org_ and *DW*_adj_ → *NFW*/*GW*^7^. Except for *GW* → *NFW*, all interactions were statistically significant.

### Detecting causality in the hydrological connectivity between *NFW*, *GW,* and *DW*

**Figure 5:**
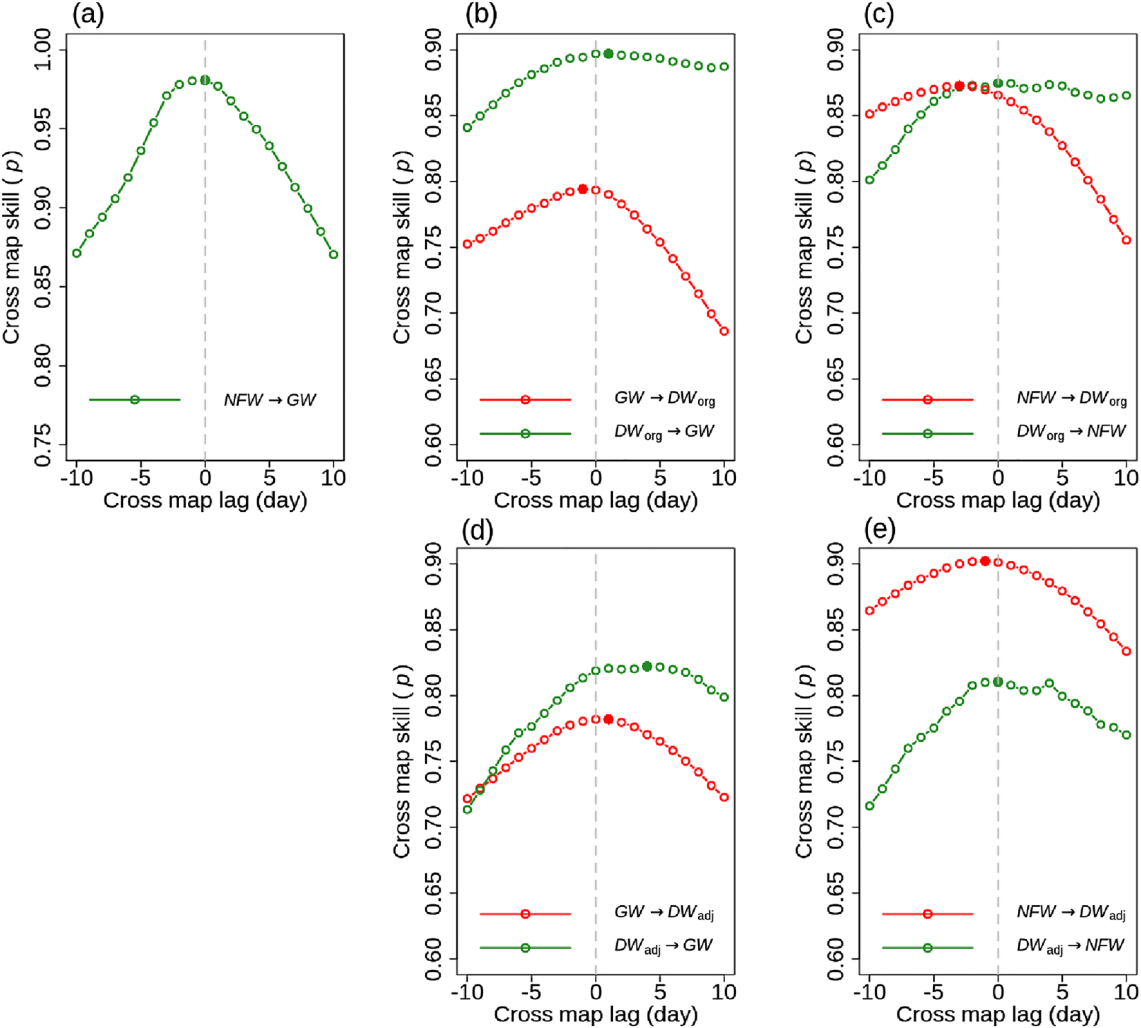
Cross map skill (*p*) with cross map lag between: (**a**) *NFW* and *GW*, (**b**) *GW* and *DW*_org_, (**c**) *NFW* and *DW*_org_, (**d**) *GW* and *DW*_adj_, and (**e**) *GW* and *DW*_adj_. x → y indicates x affects y. *NFW* and *GW* indicate non-floodplain wetland and groundwater, respectively. *DW*_org_ and *DW*_adj_ are baseflow derived from streamflow measured at USGS gauge stations #01491000 and #01491500, respectively. The figure was generated by the R 3.6.1 program.

First, we applied extended CCM method to further identify the true causality among *NFW*, *GW*, *DW*_org_ and *DW*_adj_. The true causations in Fig. 4 were further explored. Figure 5a shows that the optimal cross map lags of *NFW* → *GW* was not distinguishable from zero lag time. This was likely because the spatial configuration of the well and piezometer might lead to synchronization between *NFW* and *GW*. In addition, daily measurements might not capture lagged responses of *GW* to *NFW*.

The optimal lag time of *GW* → *DW*_org_ was observed at a negative one day (Fig. 5b), indicating that changes in *GW* would be reflected in *DW*_org_ one day later. Non-causation with the positive optimal lag time was found in the opposite direction (*DW*_org_ → *GW*). The optimal lag time of *NFW* → *DW*_org_ was negative 3 days, and the opposite direction was zero (Fig. 5c). The results of *NFW* → *DW*_org_ informed that after 3 days, any changes in *NFW* would affect *DW*_org_. Regarding the lag time in the causal chain of *NFW*, *GW*, and *DW*_org_, the CCM results represented changes in *NFW* caused changes in *GW* and then subsequently affected *DW*_org_. The lag times of among *NFW*, *GW*, and *DW*_org_ agreed with our assumption as the sum of the lag times of two direct causal links (*NFW* → *GW* and *GW* → *DW*_org_) was lower than the indirect causal link (the optimal lag of *NFW* → *DW*_org_). Moreover, the two lagged interactions (*GW* → *DW*_org_ and *NFW* → *DW*_org_) were found to be statistically significant (*p-value* < 0.01). Regarding the optimal lag times, the significance of two causal links (*GW* at t → *DW*_org_ at t + 1 and *NFW* at t → *DW*_org_ at t + 3, where t represents the time) were assessed using the method proposed by Bonotto et al.^28^ and Ye et al.^34^. The results from extended CCM well demonstrated the causal chain from *NFW* to *DW*_org_ via *GW* with different lag times.

In the case of *GW* → *DW*_adj_, the optimal lag time was a positive one day, representing that *DW*_adj_ responded to *GW* although any changes did not take place at *GW* (Fig. 5d). The optimal lag time of *DW*_adj_ → *GW* with the positive optimal lag time also showed non-causation. Interestingly, a negative one day was the optimal lag time of *NFW* → *DW*_adj_, indicating the changes in *NFW* will be reflected in *DW*_adj_ after one day. Regarding a transitive causal chain from *NFW* to *DW* through *GW*, the negative one-day optimal lag time of *NFW* → *DW*_adj_ could not support the *GW* hydrologic connectivity between *NFW* and *DW*_adj_ because *GW* was not causal to *DW*_adj_ (Fig. 5e). Therefore, the extended CCM analysis showed that the evidence of causal relationships in a transitive causal chain from *NFW* to *DW* through *GW* was only found at *DW*_org_ and not at *DW*_adj_ based on the lagged responses among them.

### Discussions and limitations

In this study, CCM was used to find causal relationships in nonlinear dynamical systems for observables with weak or moderate coupling. Previous studies explored various causal relationships between two entities in environmental systems using CCM^22–28^. Among them, different causal methods including CCM were compared for the hydrologic variable^26–28^. A study by Bonotto et al.^28^ tested the impacts of data seasonality, sampling frequency, and long-term trends on the performance of CCM. Following previous studies, this study could provide additional insight on the use of CCM on the causality between one small casual variable (i.e., *NFW*) and an aggregated affected-variable (i.e., *DW*). *NFW* is one of water storages that drains to *DW* known as the summation of intra-watershed processes, and thus *NFW* might have trivial impacts on *DW*. To partially address the uncertainty on the causal impacts of *NFW* on *DW*, this study introduced pseudo *DW* to demonstrate the reliability of CCM results. This method could offer the potential way of using CCM to see causality between a small causal variable and the aggregated-affected variable. However, the observed causal relationship between *NFW* and *DW* was uncertain although this study adopted pseudo *DW* to partially address this issue. Extensive observations along the hydraulic gradient from *NFW* to *DW* might offer reliable evidence of causation, but this is challenging. To achieve dependable causality, efforts to install multiple observations along a hydrologic gradient between *NFW* and *DW* would be critical.

This study used daily time series of *NFW*, *GW* and *DW* mainly due to missing data in *NFW* (see the green line in Fig. 3a). In-situ observational data from a well and piezometer inevitably includes missing data due to uncontrollable environment conditions. The CCM results with daily time series represented the synchronized behaviors between *NFW* and *GW* (Fig. 5a), but sub-daily time series might show the lagged interaction between *NFW* and *GW*. The CCM analysis could not be performed with missing data. Accordingly, 15-min monitoring data were converted into daily data to replace missing data by the simulations from a process-based model that demonstrated decent performance measures in this region^33^. The process-based model only simulated daily dynamics of *NFW*^33^. Sub-daily observations data are recommended to see lagged responses of *GW* to *NFW* since the CCM results were sensitive to the sampling frequency^28^.

**Figure 6:**
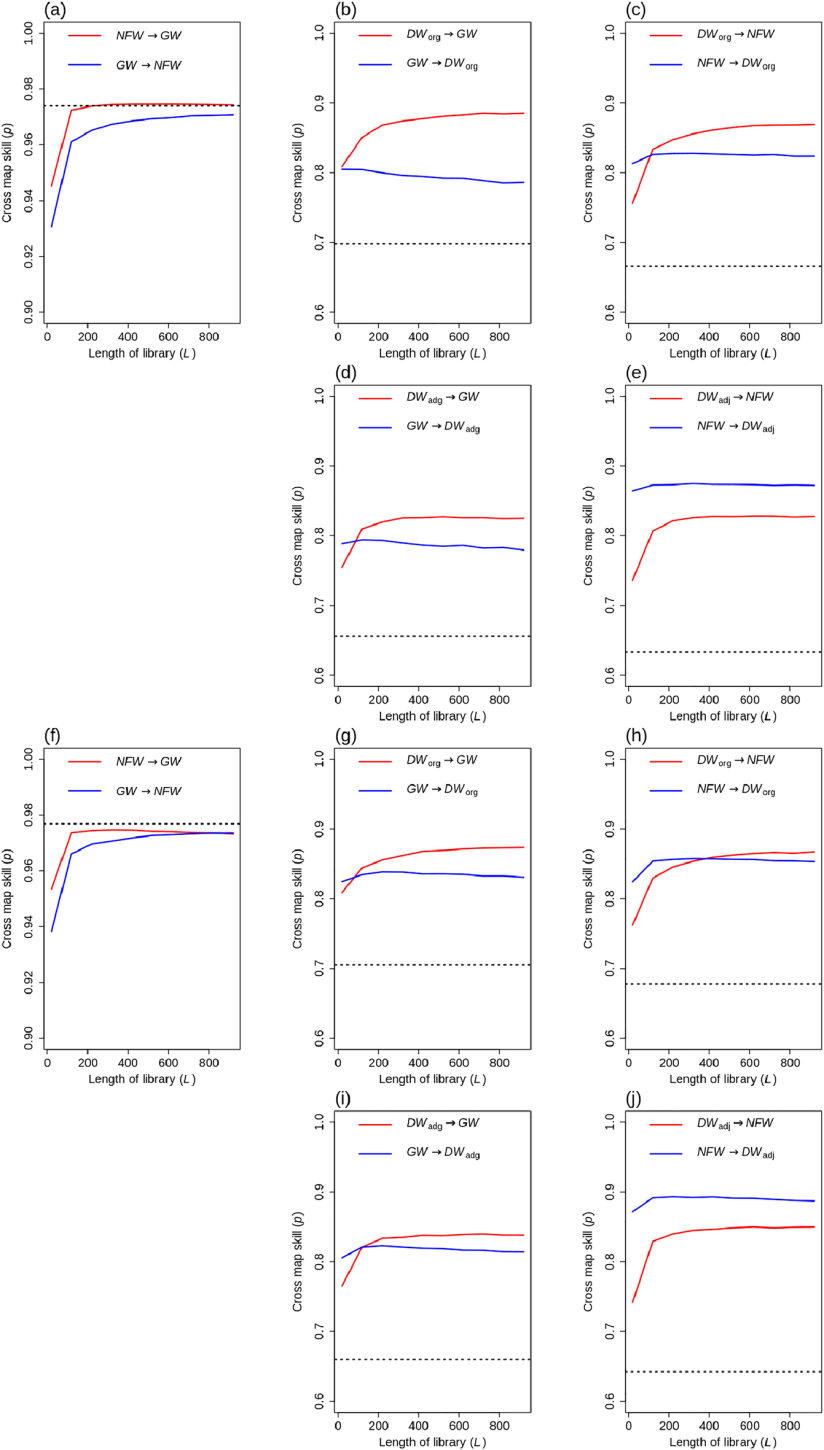
Cross map skill (*p*) of observed and estimated values as a function of the length of the library (*L*) with 3-day (**a–e**) and 7-day (**f–j**) data. The figure was generated by the R 3.6.1 program.

To test the influence of sampling frequency on CCM results, this study applied a conversion process that transformed daily data into 3-day and 7-day intervals through the averaging of daily values (Fig. 6). Despite variations in sampling frequency, the overall trends remained consistent. The highest lagged cross-correlation, represented by the dotted horizontal line, tended to increase as the temporal frequency decreased from 1 to 7 days. Notably, the causal link between *NFW* and *GW* exhibited significance with one-day data but did not show significance with 7-day data. This discrepancy was attributed to the lower cross-map skill at the full library size, which fell below the highest lagged cross-correlation (Fig. 6f). This observation suggests that the causal interaction between *NFW* and *GW* might not be effectively captured by CCM when utilizing 7-day data, as their interactions are subtle and low-frequency data fails to depict their causal behaviors. The conversion of data from higher to lower frequencies likely resulted in smoother data patterns, allowing for the generalization of subtle behaviors observed in individual *NFW*s.

The spatial configuration of a well and piezometer also likely led to synchronization between *NFW* and *GW* (Fig. 5a). If a piezometer was distant to a well, the lagged response of *GW* to *NFW* might be observed. However, the subsurface soil characteristic greatly affected the vertical water transport from *NFW* to *GW*^30^. When groundwater flow direction was not clear, measurements from a piezometer might not be associated with those from a well away from a piezometer. Thus, implementing a piezometer at the spot distant to a well is challenging. Although *NFW* and *GW* were monitored at the same spot with different vertical ranges, that practices might be the best option to see hydrologic interactions of *NFW* with sub-surface systems.

Using pseudo *DW* (represented as *DW*_adj_), this study showed a transitive causal chain from *NFW* to *DW* via *GW* in this region. The two direct causations (*NFW* → *GW* and *GW* → *DW*_org_) had a shorter lag time than one indirect causation (*NFW* → *DW*_org_), but the one direct causation (*GW* → *DW*_org_) showed a lower correlation than indirect one (*NFW* → *DW*_org_). Extended CCM method emphasized a direct causation with shorter lag times and stronger correlation than an indirection one^21^. However, our results were not consistent with the previous study^21^. It was speculated that: (1) a lower correlation in direction causation was likely due to the monitoring spot of *GW* spatially far away from *DW*; and (2) the two entities (*NFW* and *DW*_org_) in indirect causality were directly exposure to driving forces and their behaviors might have great similarity while *GW* indirectly affected by driving forces could have less similarity.

In this region, the nutrient transport time via *GW* ranged from years to decades and this time varied by subsurface conditions^35^. Our results estimated a 3-day lag time from *NFW* to *DW* via *GW*, which is not in agreement with a previous study^35^. This could be explained by “old” water in long-term groundwater storage^36,37^. The streamflow is mainly comprised of baseflow derived from long-term groundwater storage (“old” water), and the “new” precipitation has strong impacts on streamflow at storm events^36^. The water vertical transport from *NFWs* to *GW* is the pressure head of the groundwater storage, sequentially pushing the old water from upgradient to downstream and eventually releasing “old” water closer to streamflow. As groundwater storage is influenced by various landscape components, the analysis of a 3-day lag time using only the concept of “old” water may not be adequately comprehensive. The cumulative effects of multiple landscape elements on downstream water through groundwater could lead to an extended lag time for indirect causation, compared to the total of the lag times for two direct causations. More accurate estimation of lag time would be possible using tracers^38^ and extensive monitoring stations. This study is the first attempt to corroborate *GW* hydrologic connectivity between *NFW* and *DW* using CCM with available monitoring data and showed causal signals. This finding would provide insight on the role and *NFW* at a watershed. As emphasized by Bonotto et al.^28^, environmental monitoring data are often limited and thus the interpretation of CCM results should be performed with detailed observations.

## Conclusions

Our study applied a causal inference method (CCM) to detect causality between *NFW* and *DW* through *GW* on the Coastal Plain of the CBW. The hydrologic connectivity of *NFW* with *DW* via surface runoff was often observed, but demonstrating the connectivity via *GW* is difficult because strong climatic seasonality causes all landscape components have similar behaviors. CCM could detect causality among variables in nonlinear dynamical systems using time-series observations. Using CCM, we examined the causal relationship between *NFW* and *DW* via *GW* using daily time series. The CCM results showed a transitive causal chain from *NFW* to *DW* through *GW* with shorter lag of direct causation (*NFW* → *GW* and *GW* → *DW*) relative to indirect causation (*NFW* → *DW*). However, this causal chain was not observed with pseudo *DW*. These results support the notion that *NFW* is hydrologically connected with *DW* through *GW*. These findings emphasize the important role and benefits of *NFW* in landscape hydrology.

### Supplementary Information

Supplementary Information.

## Data Availability

The datasets used and/or analyzed during the current study are available from the corresponding author upon reasonable request.

## References

[1] Lane CR, Leibowitz SG, Autrey BC, LeDuc SD, Alexander LC (2018). Hydrological, physical, and chemical functions and connectivity of non-floodplain wetlands to downstream waters: A review. J. Am. Water Resour. Assoc..

[2] Lane CR (2022). Vulnerable waters are essential to watershed resilience. Ecosystems..

[3] Cohen MJ (2016). Do geographically isolated wetlands influence landscape functions?. Proc. Natl. Acad. Sci. U.S.A..

[4] Hosen JD, Armstrong AW, Palmer MA (2018). Dissolved organic matter variations in coastal plain wetland watersheds: The integrated role of hydrological connectivity, land use, and seasonality. Hydrol. Process..

[5] Pringle C (2003). What is hydrologic connectivity and why is it ecologically important?. Hydrol. Process..

[6] van der Kamp G, Hayashi M (2009). Groundwater-wetland ecosystem interaction in the semiarid glaciated plains of North America. Hydrogeol. J..

[7] Lee S (2020). Seasonal drivers of geographically isolated wetland hydrology in a low-gradient, Coastal Plain landscape. J. Hydrol..

[8] Jones CN (2019). Modeling connectivity of non-floodplain wetlands: Insights, approaches, and recommendations. J. Am. Water Resour. Assoc..

[9] Thorslund J (2018). Solute evidence for hydrological connectivity of geographically isolated wetlands. Land Degrad. Dev..

[10] Epting SM (2018). Landscape metrics as predictors of hydrologic connectivity between Coastal Plain forested wetlands and streams. Hydrol. Process..

[11] McDonough OT, Lang MW, Hosen JD, Palmer MA (2015). Surface hydrologic connectivity between Delmarva Bay wetlands and nearby streams along a gradient of agricultural alteration. Wetlands.

[12] Sivakumar B, Singh VP (2012). Hydrologic system complexity and nonlinear dynamic concepts for a catchment classification framework. Hydrol. Earth Syst. Sci..

[13] Denver JM (2014). Nitrate fate and transport through current and former depressional wetlands in an agricultural landscape, Choptank Watershed, Maryland, United States. J. Soil Water Conserv..

[14] McLaughlin DL, Kaplan DA, Cohen MJ (2014). A significant nexus: Geographically isolated wetlands influence landscape hydrology. Water Resour. Res..

[15] Phillips PJ, Shedlock RJ (1993). Hydrology and chemistry of groundwater and seasonal ponds in the Atlantic Coastal Plain in Delaware, USA. J. Hydrol. (Amst.).

[16] Winter TC, Labaugh JW (2003). Hydrologic considerations in defining isolated wetlands. Wetlands.

[17] Lindsey BD (2003). Residence Times and Nitrate Transport in Ground Water Discharging to Streams in the Chesapeake Bay Watershed.

[18] Fenstermacher DE, Rabenhorst MC, Lang MW, McCarty GW, Needelman BA (2014). Distribution, morphometry, and land use of Delmarva Bays. Wetlands.

[19] Pyzoha JE, Callahan TJ, Sun G, Trettin CC, Miwa M (2008). A conceptual hydrologic model for a forested Carolina bay depressional wetland on the Coastal Plain of South Carolina, USA. Hydrol. Process.

[20] Sugihara G (2012). Detecting causality in complex ecosystems. Science.

[21] Ye H, Deyle ER, Gilarranz LJ, Sugihara G (2015). Distinguishing time-delayed causal interactions using convergent cross mapping. Sci. Rep..

[22] van Nes EH (2015). Causal feedbacks in climate change. Nat. Clim. Change.

[23] Clark AT (2015). Spatial convergent cross mapping to detect causal relationships from short time series. Ecology.

[24] Nakayama SI, Takasuka A, Ichinokawa M, Okamura H (2018). Climate change and interspecific interactions drive species alternations between anchovy and sardine in the western North Pacific: Detection of causality by convergent cross mapping. Fish Oceanogr..

[25] Wang Y (2018). Detecting the causal effect of soil moisture on precipitation using convergent cross mapping. Sci. Rep..

[26] Delforge D, De Viron O, Vanclooster M, Van Camp M, Watlet A (2022). Detecting hydrological connectivity using causal inference from time series: Synthetic and real karstic case studies. Hydrol. Earth Syst. Sci..

[27] Ombadi M, Nguyen P, Sorooshian S, Hsu KL (2020). Evaluation of methods for causal discovery in hydrometeorological systems. Water Resour. Res..

[28] Bonotto G, Peterson TJ, Fowler K, Western AW (2022). Identifying causal interactions between groundwater and streamflow using convergent cross-mapping. Water Resour. Res..

[29] Lee S, McCarty WG, Lang WM, Li X (2020). Overview of the USDA Mid-Atlantic regional wetland conservation effects assessment project. J. Soil Water Conserv..

[30] Lee S (2018). Effects of subsurface soil characteristics on wetland-groundwater interaction in the coastal plain of the Chesapeake Bay Watershed. Hydrol. Process..

[31] Fuka, D., Walter, M., Archibald, J., Steenhuis, T. & Easton, Z. *Package ‘EcoHydRology’*. https://cran.microsoft.com/snapshot/2017-04-21/web/packages/EcoHydRology/EcoHydRology.pdf (2015).

[32] Nathan R, McMahon T (1990). Evaluation of automated techniques for base flow and recession analyses. Water Resour. Res..

[33] Qi J (2019). A coupled surface water storage and subsurface water dynamics model in SWAT for characterizing hydroperiod of geographically isolated wetlands. Adv. Water Resour..

[34] Ye, H., Clark, A., Deyle, E. & Munch, S. *rEDM: An R Package for Empirical Dynamic Modeling and Convergent Cross Mapping*. https://ha0ye.github.io/rEDM/articles/rEDM.html.

[35] Sanford WE, Pope JP (2013). Quantifying groundwater’s role in delaying improvements to Chesapeake Bay water quality. Environ. Sci. Technol..

[36] Kirchner JW (2019). Quantifying new water fractions and transit time distributions using ensemble hydrograph separation: Theory and benchmark tests. Hydrol. Earth Syst. Sci..

[37] Hrachowitz M (2016). Transit times—The link between hydrology and water quality at the catchment scale. Wiley Interdiscip. Rev. Water.

[38] Baily A, Rock L, Watson CJ, Fenton O (2011). Spatial and temporal variations in groundwater nitrate at an intensive dairy farm in South-East Ireland: Insights from stable isotope data. Agric. Ecosyst. Environ..

