# Supplementary material for: Detecting causal relationship of non-floodplain wetland hydrologic connectivity using convergent cross mapping
Source: Sci Rep. 2023 Oct 11;13:17220. doi: 10.1038/s41598-023-44071-0 (PMC10567775; doi:10.1038/s41598-023-44071-0)
Supplement: Supplementary file 1 — Supplementary Information. [file 41598_2023_44071_MOESM1_ESM.docx]

**Supplementary Material for:**

**Detecting causal relationship of non-floodplain wetland hydrologic connectivity using convergent cross mapping**

**Corresponding author:** Sangchul Lee ([sangchul.lee84@gmail.com)](mailto:sangchul.lee84@gmail.com)) and Gregory W. McCarty (greg.mccarty@usda.gov)

Contents of this file

Tables S1-S2

Figs. S1-S3

**Table S1.** List of geospatial data used in the Fig. 2

| Data | Source | Description |
| --- | --- | --- |
| Land use map | MRLC NLCD | 30-meter resolution nationwide land use map for the year 2016 |
| USGS gauge | USGS | Daily streamflow from 2016 to 2019 |
| NCDC station | NOAA NCDC | Daily precipitation from 2016 to 2019 |
| NFW | USDA-ARS, HRSL, Beltsville, MD | Non-floodplain wetland |
| NHD | USGS | high-resolution National Hydrography Dataset |

Note: MRLC NLCD: Multi-Resolution Land Characteristics Consortium National Land Cover Database; USGS: US Geological Survey; NCDC: National Climate Data Center; NOAA: National Oceanic Atmospheric Administration; USDA-ARS, HRSL: US Department of Agriculture-Agricultural Research Service, Hydrology and Remote Sensing Laboratory; NHD: National Hydrography Dataset

**Table S2.** Descriptions of the hydrogeomorphic regions over the Chesapeake Bay watershed (Lindesey et al., 2003)

| Name | Full name | Rock type | Physiographic province |
| --- | --- | --- | --- |
| APC | Appalachian Plateau carbonate | Carbonate | Appalachian Plateau |
| APS | Appalachian Plateau siliciclastic | Siliciclastic | Appalachian Plateau |
| BR | Blue Ridge | -- | Blue Ridge |
| CPD* | Coastal Plain dissected uplands | Unconsolidated | Coastal Plain |
| CPL* | Coastal Plain lowlands | Unconsolidated | Coastal Plain |
| CPU* | Coastal Plain uplands | Unconsolidated | Coastal Plain |
| ML | Mesozoic lowland | -- | Mesozoic Lowland |
| PCA | Piedmont carbonate | Carbonate | Piedmont |
| PCR | Piedmont crystalline | Crystalline and unconsolidated | Piedmont |
| VRC | Valley & Ridge carbonate | Carbonate | Valley and Ridge |
| VRS | Valley & Ridge siliciclastic | Siliciclastic | Valley and Ridge |

**
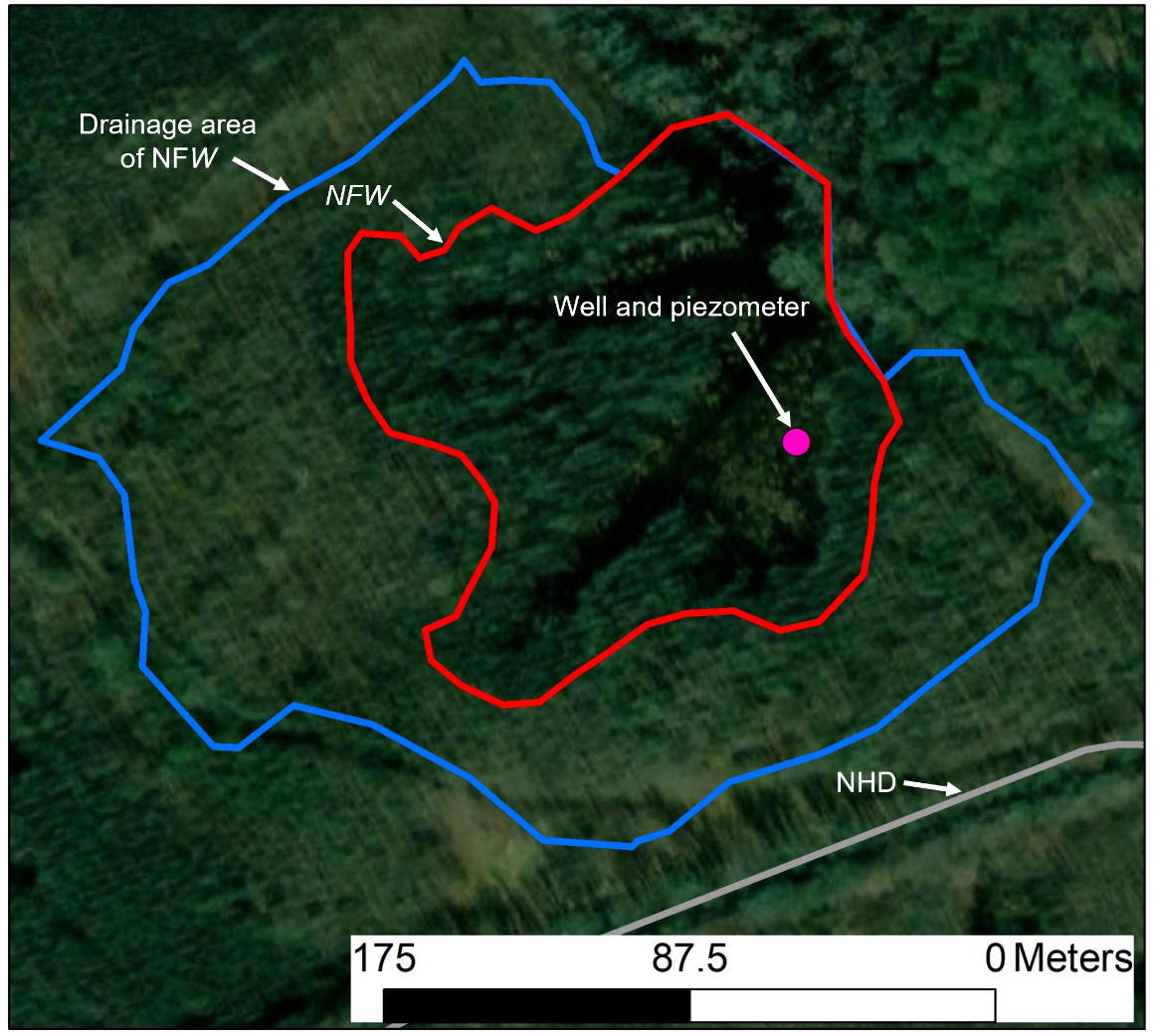
**

**Fig. S1.** The location of a well and piezometer relative to the streamline represented by National Hydrography Dataset (NHD). NFW stands for non-floodplain wetland. The map was generated by ArcMap 10.7.

**
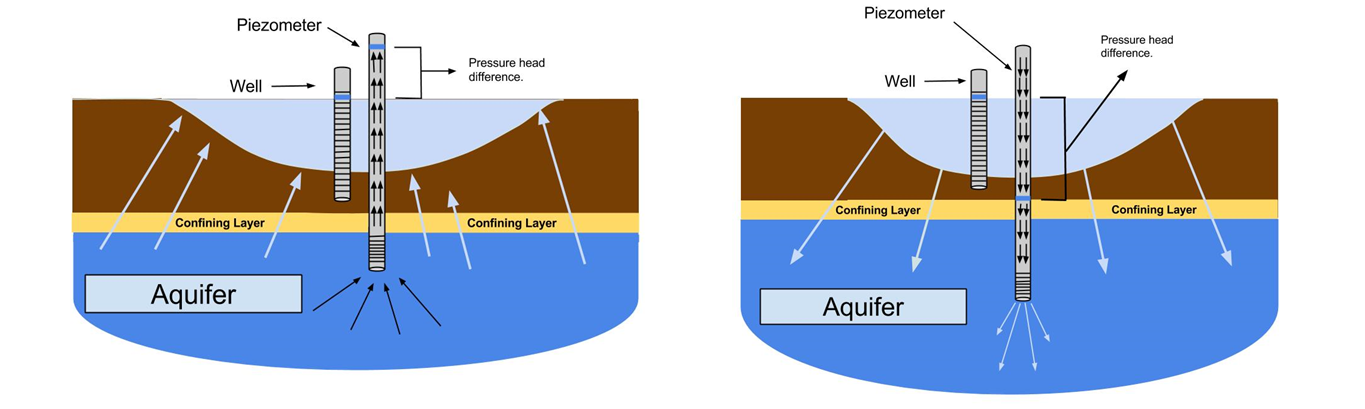
**

**Fig. S2.** The configuration of a well and piezometer to monitor non-floodplain wetland and groundwater, respectively. The figure was generated by the MS Office PowerPoint.


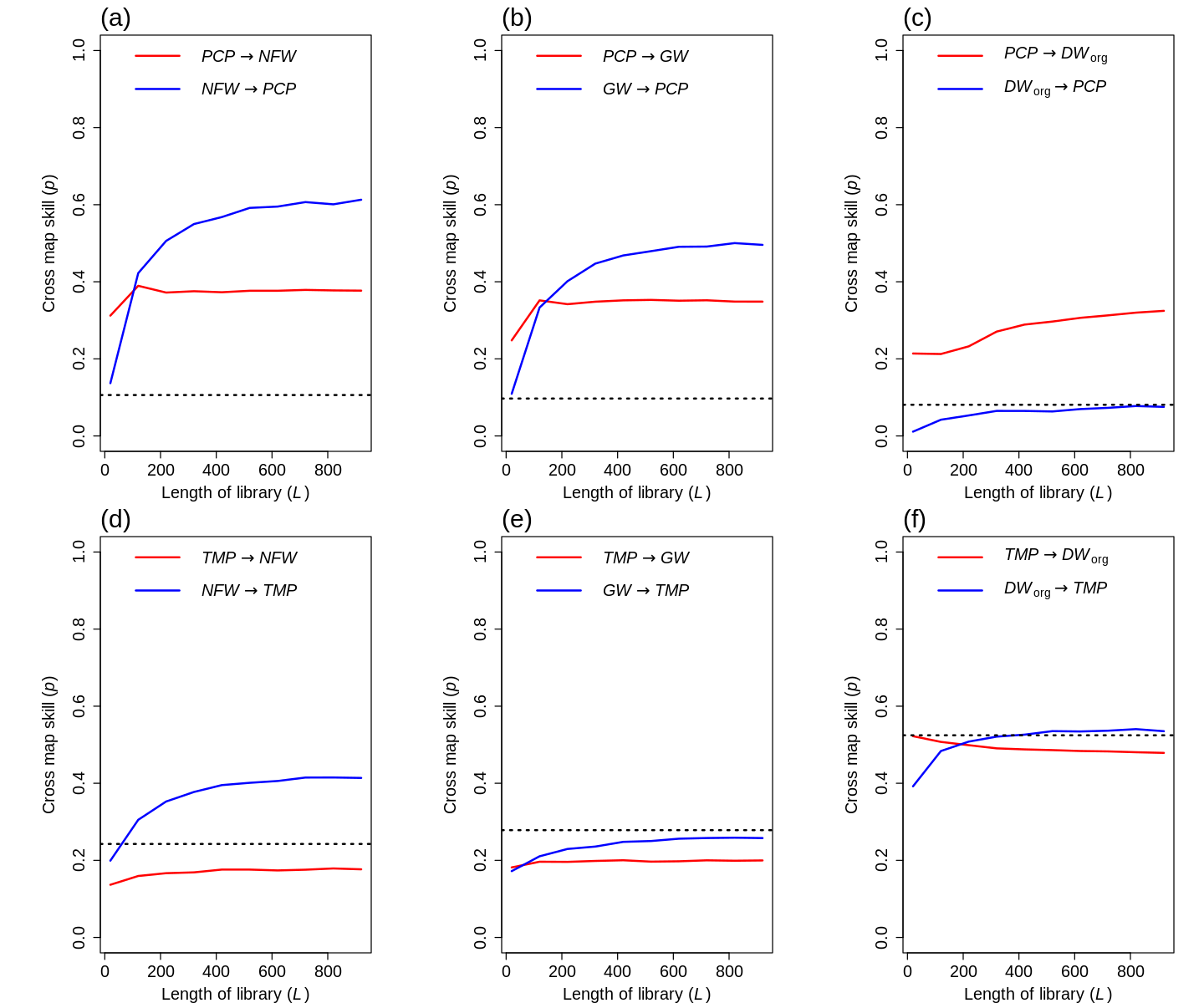


**Fig. S3.** Cross map skill (p) of observed and estimated values as a function of the length of the library (L): (a) PCP and NFW, (b) PCP and GW, and (c) PCP and DW_org._ Note: the dotted horizontal line is the highest lagged cross-correlation. x → y indicates x affects y. NFW and GW indicate non-floodplain wetland and groundwater, respectively. DW_org_ is baseflow derived from streamflow measured at USGS gauge stations #01491000, and PCP is precipitation. The figure was generated by the R 3.6.1 program.

**References**

Lindsey, B. D. *et al.* Residence Times and Nitrate Transport in Ground Water Discharging to Streams in the Chesapeake Bay Watershed.
